# Factors affecting pregnancy outcomes in young women treated with fertility-preserving therapy for well-differentiated endometrial cancer or atypical endometrial hyperplasia

**DOI:** 10.1186/s12958-015-0136-7

**Published:** 2016-01-15

**Authors:** Osamu Inoue, Toshio Hamatani, Nobuyuki Susumu, Wataru Yamagami, Seiji Ogawa, Takashi Takemoto, Akira Hirasawa, Kouji Banno, Naoaki Kuji, Mamoru Tanaka, Daisuke Aoki

**Affiliations:** Department of Obstetrics and Gynecology, Keio University School of Medicine, 35 Shinanomachi Shinjuku-ku, Tokyo, 1608582 Japan; Department of Obstetrics and Gynecology, Tokyo Medical College, 1 − 7 − 6 Nishishinjuku Shinjuku-ku, Tokyo, 1600023 Japan

**Keywords:** High-dose medroxyprogesterone acetate, MPA, Fertility-preserving therapy, Well-differentiated endometrial cancer, Atypical endometrial hyperplasia, Infertility treatment, Assisted reproductive technology

## Abstract

**Background:**

Patients hoping to preserve their fertility receive conservative treatment with high-dose medroxyprogesterone acetate (MPA) for well-differentiated endometrioid adenocarcinoma (EC) or atypical endometrial hyperplasia (AEH) . Such treatment generally involves frequent intrauterine operations, including dilation and curettage (D&C) and endometrial biopsy (EMB), which could result in endometritis, endometrial thinning, or intrauterine adhesion. In turn, any of these outcomes could adversely affect implantation and pregnancy development. The current study thus aimed to identify factors that might affect pregnancy following conservative treatment by MPA.

**Methods:**

We compared a pregnancy group (45 patients) with a non-pregnancy group (53 patients) of MPA-treated patients to evaluate the factors affecting clinical pregnancy establishment. We undertook a multivariate logistic regression analysis based on factors shown by univariate analysis to be significantly different between the groups. Univariate analysis identified number of D&C, endometrial thickness, duration of MPA administration, age of pregnancy permission (the age at which a patient was first allowed to attempt pregnancy after disappearance of the lesion), period of disappearance of lesions, and recurrence as independent variables.

**Results:**

The odds ratios (95 % confidence interval) of multivariate analysis for disease recurrence, endometrial thickness during ovulation, and age of pregnancy permission were 0.283 (0.102–0.785), 1.677 (1.251–2.248), and 0.889 (0.792–0.998), respectively. There was no significant difference in the other independent variables between groups.

**Conclusions:**

We identified three factors considered to affect pregnancy establishment following conservative treatment with MPA: recurrence, endometrial thickness during ovulation, and the age of the pregnancy permission. Introduction of infertility treatment including assisted reproductive technology (ART) soon after achieving tumor disappearance by MPA would therefore be beneficial for patients with disease recurrence, thin endometrium, or a higher age of pregnancy permission.

**Electronic supplementary material:**

The online version of this article (doi:10.1186/s12958-015-0136-7) contains supplementary material, which is available to authorized users.

## Background

Endometrial cancer (EC) is typically a disease of postmenopausal women, but approximately 5.5 % of cases occur in women younger than 40 years [1]. Of note, a trend for increasing prevalence of EC in younger patients has recently emerged [2], with cases in women younger than 40 years increasing from 45 to over 157 in a million during a 20-year period in Japan [3]. Atypical endometrial hyperplasia (AEH) is a precancerous lesion, but 29 % of such cases progress to EC within several years [4]. The standard treatment of EC/AEH includes a total hysterectomy with bilateral oophorectomy resulting in total loss of fertility [5]; however, with the recent trend for delayed marriage, many women who have been diagnosed with EC/AEH do not accept the standard treatment. Instead, conservative treatment using medroxyprogesterone acetate (MPA) is increasingly used as an effective fertility-preserving therapy for early stage EC and AEH without myometrial invasion or extra-uterine spread.

MPA confers a progesterone receptor-mediated anti-tumor effect, inhibition against estrogen action [6], and inhibition of angiogenesis not mediated via progesterone receptors [7]. MPA may also reduce the number of glandular cells and decidualization of the stroma [6, 8]. A disadvantage of the conservative treatment with MPA is the need for frequent intrauterine operations including dilation and curettage (D&C) and endometrial biopsy (EMB) that can cause endometritis, endometrial thinning, and intra-uterine adhesion. These potential effects of MPA raise concerns about associated adverse effects on uterine implantation, as does the high tumor relapse rate associated with such conservative treatment. Therefore, patients treated by MPA are advised to try pregnancy establishment early after the treatment.

Pregnancy is commonly reported following conservative treatment [9–13]; however, there are no reports on the factors that contribute to pregnancy in such patients. In the current study, we compared a pregnancy group with a non-pregnancy group to elucidate variables significant in establishing pregnancy after conservative treatment for EC/AEH.

## Methods

The institutional review board of Keio University School of Medicine approved the current retrospective study (approved number # 20110237), which comprised patients diagnosed with EC stage IA (G1) or AEH at the Keio University Hospital from January 1998 to December 2012. Four patients diagnosed as EC stage IA (G2) and who clearly understood the significant risks of disease recurrence and progression elected to receive conservative treatment. After careful examination using transvaginal ultrasonography, magnetic resonance imaging, and hysteroscopy to rule out myometrial or cervical invasion, and, if needed, computed tomography to confirm that the patient had neither distant metastasis nor double cancer, endometrial curettage was performed to determine the initial pathological diagnosis and to remove lesions. Informed consent was obtained from all patients regarding the risk of disease progression, in undergoing conservative treatment with MPA (600 mg/day) instead of the standard therapy of total hysterectomy with bilateral salpingo-oophorectomy. We almost performed conservative treatment according to the same protocol (Additional file 1: Figure S1). An endometrial biopsy was performed once a month up to three months after initiating MPA administration, and D&C was performed at 4 months using a metallic curette. In cases with residual lesions, MPA administration was continued, and D&C was performed every 2 months for up to 12 months to monitor any progression of the lesion. In cases with no residual lesions, MPA was discontinued and an attempt of pregnancy was permitted, but EMB was concurrently performed every 3–4 months. Patients who did not wish to conceive immediately underwent Holmstrom therapy, whereby MPA (15 mg/day) was administered orally for 12 days after 14 days from the start of withdrawal bleeding.

### Study end-points

Subjects were divided into a pregnancy group and a non-pregnancy group, and data were collected regarding number of D&C, duration of MPA administration, and endometrial thickness during ovulation. We then analyzed the data to determine the pregnancy-affecting factors.

### Exclusion criteria

All patients undergoing conservative treatment (*n =* 174) did not necessarily wish to conceive immediately; therefore, subjects were limited to those wishing to conceive following therapy. We excluded women aged ≥ 42 years at the time of the initial treatment (*n =* 11), women without a partner (*n =* 44), and women with no fertile period or for whom detailed medical records could not be obtained immediately following conservative treatment (*n =* 17). The remaining 98 women were divided into a pregnancy group (*n =* 45) and a non-pregnancy group (*n =* 53).

### Definitions

In the present study, pregnancy was defined as a serum level of human chorionic gonadotropin ≥ 25 mIU/mL or observation of a gestational sac on a transvaginal ultrasound scan. An abnormal menstrual cycle was defined as a menstrual cycle not lasting 25–38 days. Endometrial thickness is reported to reach its minimum on day 4 of the menstrual cycle, then increase by 1 mm/day to a plateau on day 9 [14]. Therefore, endometrial thickness during ovulation was measured in the sagittal plane by transvaginal ultrasound scan when the follicle diameter was observed to be ≥ 16 mm. The age of pregnancy permission was defined as the age at which the patient was first allowed to attempt pregnancy after disappearance of the lesion. Periods of disappearance of lesions were defined as from the start of the administration till diagnosis of the disappearance of lesions. Follow up was defined as the period from the start of MPA administration to the final medical examination. Possible intrauterine adhesion was investigated in 81 cases in which hysteroscopy was performed during MPA therapy and in which a detailed intrauterine examination was performed during treatment by transcervical resection. A good-quality embryo was morphologically defined by a Veeck classification ≥ grade 2 on day 2/3 [15] or a Gardner classification ≥ 3BB on day 5 after oocyte pick up [16].

### Statistical analysis

Shapiro-Wilk tests for all data showed a non-normal distribution. Mann–Whitney U tests were used for statistical analysis, while nominal scale comparisons were performed using the Pearson Chi-squared test (see Footnotes a and b in Table 1). Multicollinearity was examined using the Spearman rank correlation coefficient to create a correlation matrix; it was confirmed that no combination of the independent variables demonstrated a correlation coefficient with an absolute value ≥ 0.9. Multivariate analysis was performed in the pregnancy and non-pregnancy groups using endometrial thickness, number of D&C, duration of MPA administration, age of pregnancy permission, and recurrence as independent variables, using a logistic regression to determine the likelihood ratio. Model Chi-squared test results with *P* < 0.05 were considered to be statistically significant. Analyses were conducted using SPSS version 15.0 (SPSS Inc., Chicago, IL, USA).

## Results

### Patient characteristics

Patient characteristics of the pregnancy group (*n =* 45) and the non-pregnancy group (*n =* 53) are summarized in Table 1. The average age of the first pregnancy was 34.8 ± 3.9 years. There were no significant differences between the groups in age of initial treatment, nulliparity, histological type/grade, body mass index, PCO on ultrasonography, irregular menstrual cycle, or use of ovarian stimulation.

**Table 1:** Patient characteristics

	Total	Pregnancy	Non-pregnancy	*P*
Patients (n)	98	45	53	-
Age of initial treatment (years)	33.8 ± 4.4	32.8 ± 4.6	34.6 ± 4.1	0.051^a^
Pregnancy age (years)	-	34.8 ± 3.9	-	-
Nulliparity (%)	87 (88.8)	42 (83.3)	52 (92.9)	0.088^a^
Histological type/grade				0.408^b^
AEH	37	15	22	
G1/G2	61	30	31	
BMI (kg/m2)	21.9 ± 4.7	21.5 ± 4.9	22.3 ± 4.5	0.264^a^
PCO on ultrasonography (%)	29 (29.6)	16 (35.6)	13 (24.5)	0.233^a^
Irregular menstrual cycle (%)	54 (55.1)	24 (53.3)	30 (56.6)	0.116^a^
Use of ovarian stimulation (%)	58 (59.2)	30 (66.7)	28 (52.8)	0.165^a^

Note : Values listed as mean ± standard deviation

^a^Pregnancy versus Non-pregnancy (Mann–Whitney *U* test)

^b^Pregnancy versus Non-pregnancy (Chi-squared test)

### Infertility treatments after conservative treatment

Table 2 shows that 68.4 % of the pregnancies resulted from infertility treatment including timing treatment, controlled ovarian stimulation, IUI, and *in vitro* fertilization (IVF)/intracytoplasmic sperm injection (ICSI) (22.8 %), but 71.9 % of them were achieved by natural insemination. With respect to the non-pregnancy group, 84.9 % of the subjects also had the aid of infertility treatment, and 37.7 % of them underwent IVF.

**Table 2:** Infertility treatment rates in the pregnancy and the non-pregnancy groups

	Pregnancy	Non-pregnancy
Natural insemination (%)	41 (71.9)	28 (52.8)
No infertility treatment (%)	18 (31.6)	8 (15.1)
Timing treatment (%)	8 (14.0)	8 (15.1)
Clomifene Citrate-timing treatment (%)	8 (14.0)	6 (11.3)
HMG-timing treatment (%)	7 (12.3)	6 (11.3)
IUI (%)	3 (5.3)	5 (9.5)
IVF/ICSI (%)	13 (22.8)	20 (37.7)
Total	57 (100)	53 (100)

### Matters relating to conservative treatment

The duration of MPA administration was significantly shorter in the pregnancy group at 277.5 ± 167.0 days than in the non-pregnancy group at 431.5 ± 342.5 days, as was the time to disappearance of lesions (136.2 ± 133.8 days vs. 187.0 ± 147.7) (Table 3). The age of pregnancy permission and the number of D&C procedures performed were also both significantly lower in the pregnancy group than in the non-pregnancy group (33.9 ± 4.4 years vs. 36.0 ± 4.8 years; 4.18 ± 2.34 vs. 5.66 ± 3.77, respectively). Endometrial thickness during ovulation was significantly higher in the pregnancy group at 8.56 ± 1.87 mm than in the non-pregnancy group at 6.70 ± 1.87 mm.

**Table 3:** Matters relating to conservative treatment

	Total	Pregnancy	Non-pregnancy	P*
Age of pregnancy permission	35.0 ± 4.7	33.9 ± 4.4	36.0 ± 4.8	0.023
Number of D&C (times)	4.98 ± 3.27	4.18 ± 2.34	5.66 ± 3.77	0.049
Endometrial thickness during ovulation (mm)	7.50 ± 2.08	8.56 ± 1.87	6.70 ± 1.87	<0.001
Duration of MPA administration (days)	357.7 ± 285.4	277.5 ± 167.0	431.5 ± 342.5	0.010
Periods of disappearance of lesions (days)	164.0 ± 143.1	136.2 ± 133.8	187.0 ± 147.7	0.042
Periods from the last MPA administration to menstruation or starting date of the pregnancy (days)		314.0 ± 392.8	ー	ー
Infertile period after conservative treatment (days)	1146 ± 1024	1206 ± 887	1096 ± 1131	0.116
Follow up (days)	2032 ± 1266	2027 ± 1115	2037 ± 1389	0.592
Recurrence (%)	61 (62.2)	23 (51.1)	38 (71.7)	0.036
Intrauterine adhesion (%)	20/81 (24.7)	5/29 (17.2)	15/52 (28.8)	0.191

Note : Values listed as mean ± standard deviation

*Pregnancy versus Non-pregnancy (Mann–Whitney *U* test)

Univariate analysis showed significant differences between the pregnancy and the non-pregnancy groups in number of D&C, endometrial thickness, duration of MPA administration, age of pregnancy permission, and recurrence. Thus, these five factors were used as independent variables for the multivariate logistic regression analysis (Table 4), which identified recurrence (odds ratio 0.283; 95 % CI 0.10–0.785), endometrial thickness during ovulation (1.677; 1.251–2.248), and age of pregnancy permission (0.889; 0.792–0.998) as significant factors affecting pregnancy outcomes.

**Table 4:** Logistic regression analysis

	*P*	Odd ratio	95 % CI
Endometrial thickness	0.001	1.677	1.251–2.248
Recurrence	0.015	0.283	0.102–0.785
Age of pregnancy permission	0.046	0.889	0.792–0.998
Duration of MPA administration	0.065		
Number of D&C	0.407		
Periods of disappearance of lesions	0.358		

### Number of D&C as a potential confounder of endometrial thickness

**Fig. 1:**
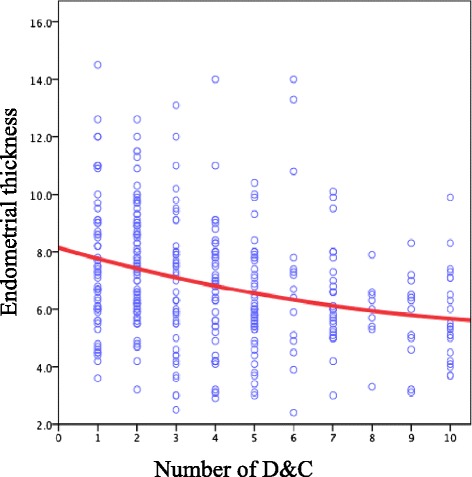
Regression analysis of the correlation between ultrasonographically measured endometrial thickness during 365 ovulation cycles (included the same person) and the number of D&C procedures (1–10) for all the subjects yielded an absolute value of 0.4, indicating a correlation. The regression equation was significant (*P* < 0.01) according to an analysis of variance (ANOVA) table, as was the regression coefficient (*P* < 0.01). Curvilinear regression demonstrated that endometrial thickness during ovulation decreases as the number of D&C procedures increase

Although few studies have reported on the role of D&C in the etiology of thin endometrium [17], the relationship between these variables remains controversial. In the current study, regression analysis of the correlation between ultrasonographically measured endometrial thickness during 365 ovulation cycles (included the same person) and the number of D&C procedures (1–10) for all the subjects yielded an absolute value of 0.4, indicating a correlation (Fig. 1). In the regression analysis, the regression equation was significant (*P* < 0.01) according to an analysis of variance (ANOVA) table, as was the regression coefficient (*P* < 0.01). Curvilinear regression demonstrated that endometrial thickness during ovulation decreases as the number of D&C procedures increase.

## Discussion

In the current retrospective study, the EC/AEH patients who had undergone conservative treatment with MPA were divided into pregnancy and non-pregnancy groups, which were compared to determine factors in conservative treatment contributing to pregnancy after achieving complete response. Based on the multivariate multiple regression analysis, disease recurrence, endometrial thickness during ovulation, and the age of pregnancy permission were significantly associated with pregnancy establishment. Although a significant difference in the age of pregnancy permission was observed, with an OR of 0.889, its effect on pregnancy was considered small. Thus, we concluded that recurrence and endometrial thickness, with ORs of 0.283 and 1.677, respectively, have an important effect on pregnancy establishment among the factors examined in the study. In general, sufficient endometrial thickness is important for pregnancy establishment [18–25], and when endometrial thickness is ≤ 7 mm, the functional layer is thin or absent, resulting in implantation of the embryo near the spinal artery. High blood flow impedance of radial arteries impairs the growth of the glandular epithelium and results in decreased vascular endothelial growth factor (VEGF) levels in the endometrium. Low VEGF and low blood flow lead to a “thin” endometrium, which is related to impaired endometrial receptivity [26]. These previous data thus support the result of our multivariate multiple regression analysis that endometrial thickness is an important factor for pregnancy outcomes after conservative treatment for EC/AEH. Therefore, it is necessary to minimize damage to normal endometrium. To this end, Fujimoto et al. [27, 28] suggested that hysteroscopic resection of early stage EC combined with hormonal therapy might be more effective than endometrial curettage for eliminating lesions in such patients hoping to preserve their fertility. Such an approach would minimize a specimen, but does not increase the risk of peritoneal dissemination [29].

We further studied if the number of D&C procedures is a potential confounder of thin endometrium. Endometrial thickness was measured in 365 cycles of ovulation following conservative treatment and regression analysis consequently showed that endometrial thickness during ovulation decreases as the number of D&C increases (*P* < 0.01). Furthermore, repeated D&C procedures might increase intrauterine adhesion [30]. In the current study, the frequency of intrauterine adhesions after D&C in the pregnancy group was not significantly different from that in the non-pregnancy group by univariate analysis. Although nearly all of the intrauterine adhesions that we observed were mild, film-like adhesions not likely to affect pregnancy, a future extensive analysis of more cases is needed to accurately determine the number of D&C likely to significantly impair fertility. On the other hand, MPA administration causes histological changes in the endometrium, including a decreased gland-to-stroma ratio, reduction in the number of glandular cells, decidualization of the stroma, and decreased mitosis [6, 8]. These effects may cause atrophy and thinning of the endometrium, and considerable time is likely needed for the tissue’s functional recovery after MPA termination. Randall et al. [31] reported that a median duration of 9 months is required after progestin therapy for the lesions to improve, and herein, we showed that the period from MPA termination till the beginning of the final menstrual cycle before pregnancy was 314.0 ± 392.8 days. Accordingly, the duration of MPA administration also might be a potential confounder of endometrial thinning.

Conservative treatment needs to provide a promising option for EC/AEH patients who wish to preserve fertility. However, there is a considerable risk of lesion recurrence and disease progression with such therapies. Yamazawa et al. [32] reported a recurrence rate of approximately 35 %, while Ushijima and Chiva et al. [33, 34] reported that tumors recur in 20–47.9 months. Furthermore, the current study found that 61 of 98 (62.2 %) patients experienced recurrence during the follow-up period (2189 ± 1346 days) from the start of MPA administration to the final medical examination. Therefore, pregnancy should be promptly attempted following complete response to conservative treatment in EC/AEH patients. Furthermore, it is known that the infertility rate is higher among EC/AEH patients than among the general population, due to an increased prevalence of anovulatory cycles or polycystic ovaries. Based on these discussion points, infertility treatment is strongly recommended after achieving a complete response to conservative treatment, to enhance the chance of pregnancy. All patients tried to conceive by natural conception after receiving MPA, and those who could not easily fall pregnant proceeded to IUI or IVF. The current study indeed demonstrated many cases of pregnancy occurring early after the cessation of treatment through natural methods (no infertility treatment: 18 cases, timed sexual intercourse: 8 cases) and the use of fertility agents for ovarian stimulation (clomiphene citrate: 8 cases, HMG: 7 cases). In addition, but importantly, Ichinose et al. [35] reported that the use of fertility drugs did not increase the recurrence of EC/AEH.

ART has been reported as a useful alternative for EC/AEH patients after conservative treatment [36]; however, in the current study, only 13 out of 52 pregnancies required the aid of ART, and only 2 of the 15 patients who received ART at our hospitals in the study were successful in achieving pregnancy (Additional file 2: Table S1 and 3: Table S2). These patients who became pregnant were aged 32 and 34 years, and did not have thin endometrium. The other 13 non-pregnant patients were at least 37 years old, and 9 were older than 40 years. Since morphologically good embryos were transferred in the majority of cases based on the reproductive outcomes achieved in another clinic, we deduced that failed IVF was most likely attributable to an endometrial factor and not embryo quality. At an advanced age, endometrial thinning resulted from MPA therapy and D&C, as well as maternal age, could contribute to poor pregnancy outcomes with ART. Thus, this study could not provide any evidence on the effectiveness of ART for EC/AEH patients after conservative treatment. Nonetheless, the age of pregnancy permission and recurrence were also shown by the multivariate regression analysis as a significant factor for pregnancy outcomes after conservative treatment. A higher age of pregnancy permission and a prolonged period of medical treatment due to recurrence may be accompanied by ovarian aging and hypofunction. Considering the possibility of recurrence, early referral to an infertility treatment specialist and proactive recommendation of ART should be advised for EC/AEH patients who have already experienced recurrence, have a long period till remission, or have a higher age of pregnancy permission and/or thin endometrium.

## Conclusions

Tumor recurrence, endometrial thickness during ovulation, and the age of pregnancy permission were found to affect pregnancy establishment following conservative treatment. A prompt introduction of infertility treatment including ART following conservative treatment should be advised for EC/AEH patients with thin endometrium, a higher age of pregnancy permission or recurrence, and a long period till remission.

## Additional files

Additional file 1: Figure S1. Our conservative treatment protocol. We generally performed conservative treatment according to the same protocol. An endometrial biopsy was performed once a month up to three months after initiating MPA administration and D&C was performed at 4 months. In cases with residual lesions, MPA administration was continued, and D&C was performed every 2 months for up to 12 months. In cases with no residual lesions, MPA was discontinued and an attempt at pregnancy was permitted, while EMB was concurrently performed every 3–4 months. (PPTX 70 kb) Additional file 2: Table S1. Baseline characteristics for IVF cases in Keio University. (PPTX 63 kb) Additional file 3: Table S2. Reproductive outcomes. (PPTX 68 kb)
