# Supplementary material for: Factors affecting pregnancy outcomes in young women treated with fertility-preserving therapy for well-differentiated endometrial cancer or atypical endometrial hyperplasia
Source: Reprod Biol Endocrinol. 2016 Jan 15;14:2. doi: 10.1186/s12958-015-0136-7 (PMC4714532; doi:10.1186/s12958-015-0136-7)
Supplement: Additional file 1: Figure S1. — Our conservative treatment protocol. We generally performed conservative treatment according to the same protocol. An endometrial biopsy was performed once a month up to three months after initiating MPA administration and D&C was performed at 4 months. In cases with residual lesions, MPA administration was continued, and D&C was performed every 2 months for up to 12 months. In cases with no residual lesions, MPA was discontinued and an attempt at pregnancy was permitted, while EMB was concurrently performed every 3–4 months. (PPTX 70 kb) [file 12958_2015_136_MOESM1_ESM.pptx]

## Slide 1
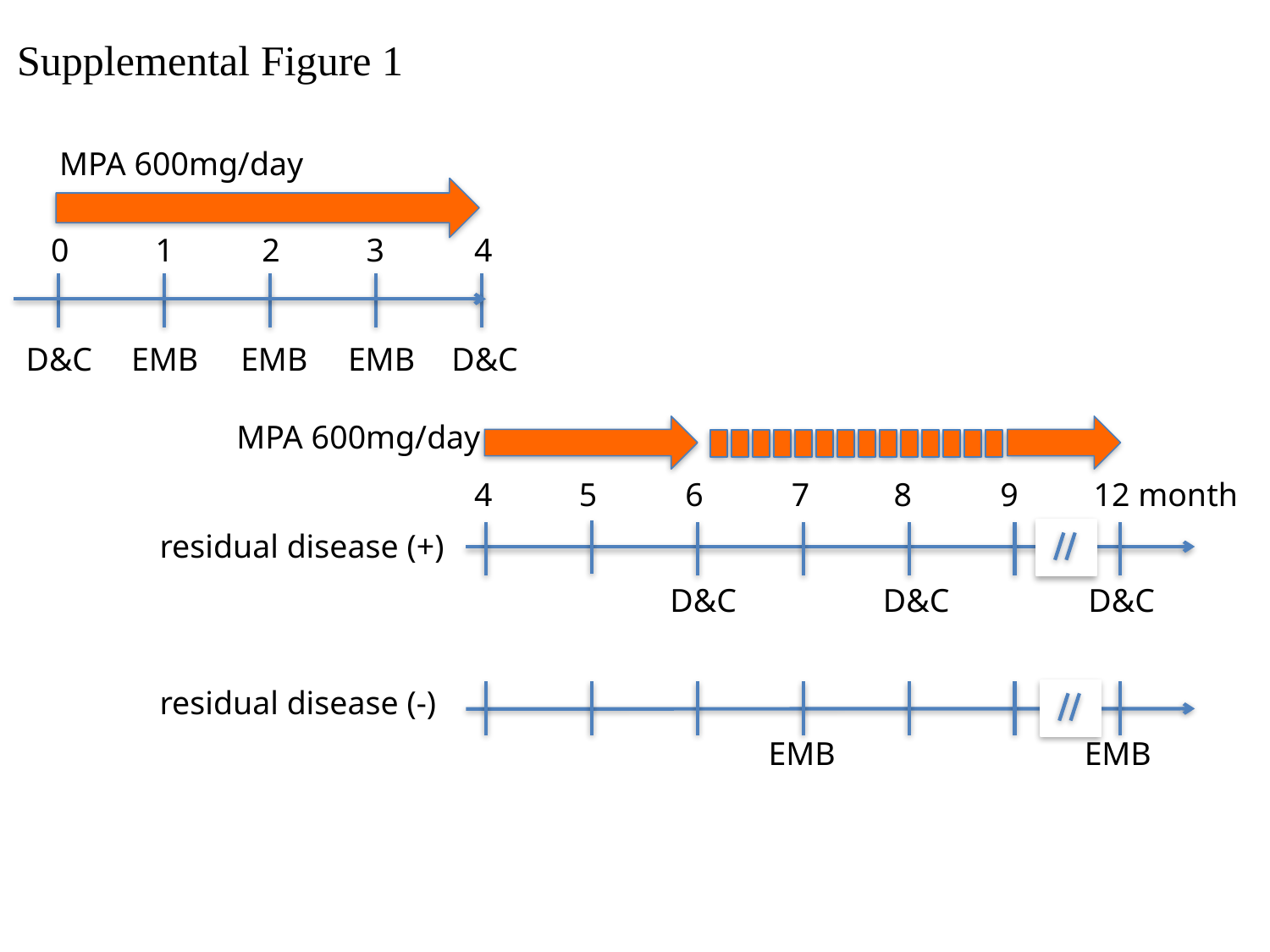

Supplemental Figure 1
MPA 600mg/day
0
1
2
3
4
D&C
EMB
EMB
EMB
D&C
MPA 600mg/day
4
5
6
7
8
9
12 month
residual disease (+)
D&C
D&C
D&C
residual disease (-)
EMB
EMB
