# Supplementary material for: Factors affecting pregnancy outcomes in young women treated with fertility-preserving therapy for well-differentiated endometrial cancer or atypical endometrial hyperplasia
Source: Reprod Biol Endocrinol. 2016 Jan 15;14:2. doi: 10.1186/s12958-015-0136-7 (PMC4714532; doi:10.1186/s12958-015-0136-7)
Supplement: Additional file 2: Table S1. — Baseline characteristics for IVF cases in Keio University. (PPTX 63 kb) [file 12958_2015_136_MOESM2_ESM.pptx]

## Slide 1
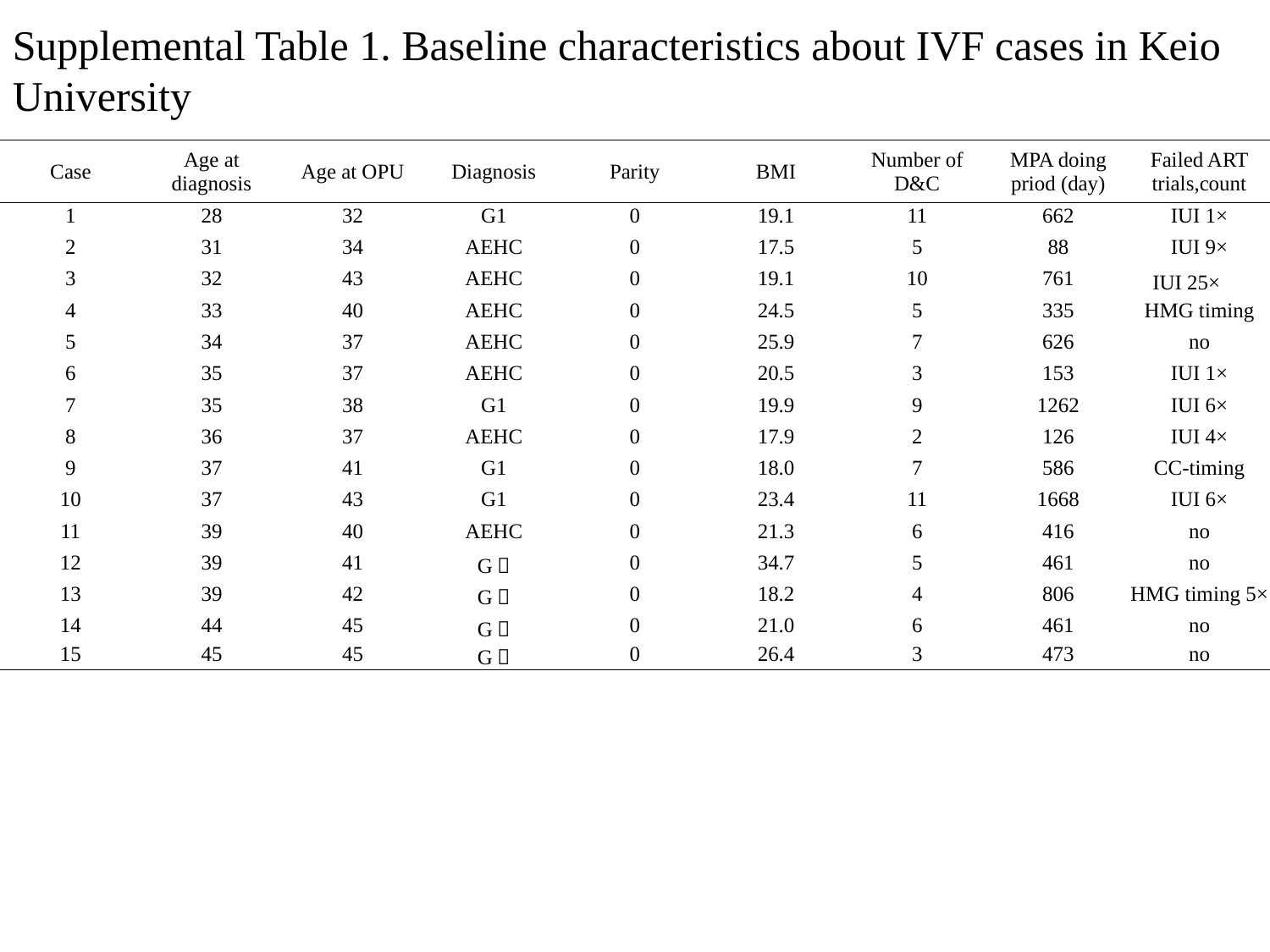

Supplemental Table 1. Baseline characteristics about IVF cases in Keio University
| Case | Age at diagnosis | Age at OPU | Diagnosis | Parity | BMI | Number of D&C | MPA doing priod (day) | Failed ART trials,count |
| --- | --- | --- | --- | --- | --- | --- | --- | --- |
| 1 | 28 | 32 | G1 | 0 | 19.1 | 11 | 662 | IUI 1× |
| 2 | 31 | 34 | AEHC | 0 | 17.5 | 5 | 88 | IUI 9× |
| 3 | 32 | 43 | AEHC | 0 | 19.1 | 10 | 761 | IUI 25× |
| 4 | 33 | 40 | AEHC | 0 | 24.5 | 5 | 335 | HMG timing |
| 5 | 34 | 37 | AEHC | 0 | 25.9 | 7 | 626 | no |
| 6 | 35 | 37 | AEHC | 0 | 20.5 | 3 | 153 | IUI 1× |
| 7 | 35 | 38 | G1 | 0 | 19.9 | 9 | 1262 | IUI 6× |
| 8 | 36 | 37 | AEHC | 0 | 17.9 | 2 | 126 | IUI 4× |
| 9 | 37 | 41 | G1 | 0 | 18.0 | 7 | 586 | CC-timing |
| 10 | 37 | 43 | G1 | 0 | 23.4 | 11 | 1668 | IUI 6× |
| 11 | 39 | 40 | AEHC | 0 | 21.3 | 6 | 416 | no |
| 12 | 39 | 41 | G１ | 0 | 34.7 | 5 | 461 | no |
| 13 | 39 | 42 | G１ | 0 | 18.2 | 4 | 806 | HMG timing 5× |
| 14 | 44 | 45 | G１ | 0 | 21.0 | 6 | 461 | no |
| 15 | 45 | 45 | G１ | 0 | 26.4 | 3 | 473 | no |
