# Supplementary material for: Factors affecting pregnancy outcomes in young women treated with fertility-preserving therapy for well-differentiated endometrial cancer or atypical endometrial hyperplasia
Source: Reprod Biol Endocrinol. 2016 Jan 15;14:2. doi: 10.1186/s12958-015-0136-7 (PMC4714532; doi:10.1186/s12958-015-0136-7)
Supplement: Additional file 3: Table S2. — Reproductive outcomes. (PPTX 68 kb) [file 12958_2015_136_MOESM3_ESM.pptx]

## Slide 1
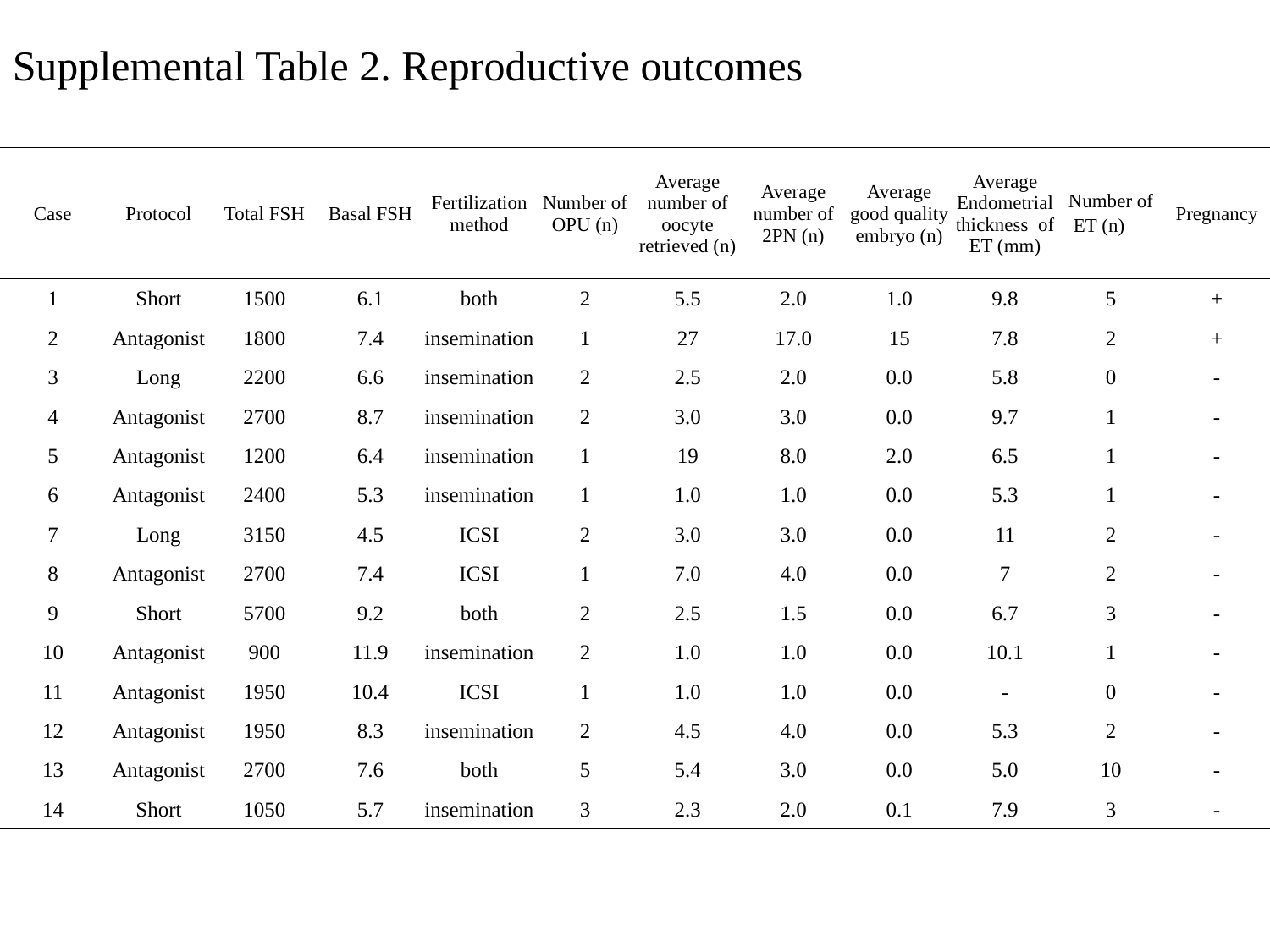

Supplemental Table 2. Reproductive outcomes
| Case | Protocol | Total FSH | Basal FSH | Fertilization method | Number of OPU (n) | Average number of oocyte retrieved (n) | Average number of 2PN (n) | Average good quality embryo (n) | Average Endometrial thickness of ET (mm) | Number of ET (n) | Pregnancy |
| --- | --- | --- | --- | --- | --- | --- | --- | --- | --- | --- | --- |
| 1 | Short | 1500 | 6.1 | both | 2 | 5.5 | 2.0 | 1.0 | 9.8 | 5 | + |
| 2 | Antagonist | 1800 | 7.4 | insemination | 1 | 27 | 17.0 | 15 | 7.8 | 2 | + |
| 3 | Long | 2200 | 6.6 | insemination | 2 | 2.5 | 2.0 | 0.0 | 5.8 | 0 | - |
| 4 | Antagonist | 2700 | 8.7 | insemination | 2 | 3.0 | 3.0 | 0.0 | 9.7 | 1 | - |
| 5 | Antagonist | 1200 | 6.4 | insemination | 1 | 19 | 8.0 | 2.0 | 6.5 | 1 | - |
| 6 | Antagonist | 2400 | 5.3 | insemination | 1 | 1.0 | 1.0 | 0.0 | 5.3 | 1 | - |
| 7 | Long | 3150 | 4.5 | ICSI | 2 | 3.0 | 3.0 | 0.0 | 11 | 2 | - |
| 8 | Antagonist | 2700 | 7.4 | ICSI | 1 | 7.0 | 4.0 | 0.0 | 7 | 2 | - |
| 9 | Short | 5700 | 9.2 | both | 2 | 2.5 | 1.5 | 0.0 | 6.7 | 3 | - |
| 10 | Antagonist | 900 | 11.9 | insemination | 2 | 1.0 | 1.0 | 0.0 | 10.1 | 1 | - |
| 11 | Antagonist | 1950 | 10.4 | ICSI | 1 | 1.0 | 1.0 | 0.0 | - | 0 | - |
| 12 | Antagonist | 1950 | 8.3 | insemination | 2 | 4.5 | 4.0 | 0.0 | 5.3 | 2 | - |
| 13 | Antagonist | 2700 | 7.6 | both | 5 | 5.4 | 3.0 | 0.0 | 5.0 | 10 | - |
| 14 | Short | 1050 | 5.7 | insemination | 3 | 2.3 | 2.0 | 0.1 | 7.9 | 3 | - |
